# Correction: Effects of graphene oxide size on curing kinetics of epoxy resin

**DOI:** 10.1039/d3ra90103c

**Published:** 2023-11-02

**Authors:** Xuebing Chen, Weijiao Jiang, Bo Hu, Zhiming Liang, Yue Zhang, Jian Kang, Ya Cao, Ming Xiang

**Affiliations:** a State Key Laboratory of Polymer Materials Engineering, Polymer Research Institute of Sichuan University Chengdu 610065 China; b Dongfang Electric Machinery Co., Ltd Deyang 618000 China metaspark@163.com

## Abstract

Correction for ‘Effects of graphene oxide size on curing kinetics of epoxy resin’ by Xuebing Chen *et al.*, *RSC Adv.*, 2021, **11**, 29215–29226, https://doi.org/10.1039/D1RA05234A.

In the original article, the authors regret an error in Fig. 6. Fig. 6 contained an error in the label for the *y* axis, which was incorrectly labelled as ln(*β*/*T*_p_^2^). A corrected Fig. 6 is shown here, with the *y* axis labelled as ‘Weight percentage (%)’ to reflect the correct TGA test curve.

**Fig. 6:**
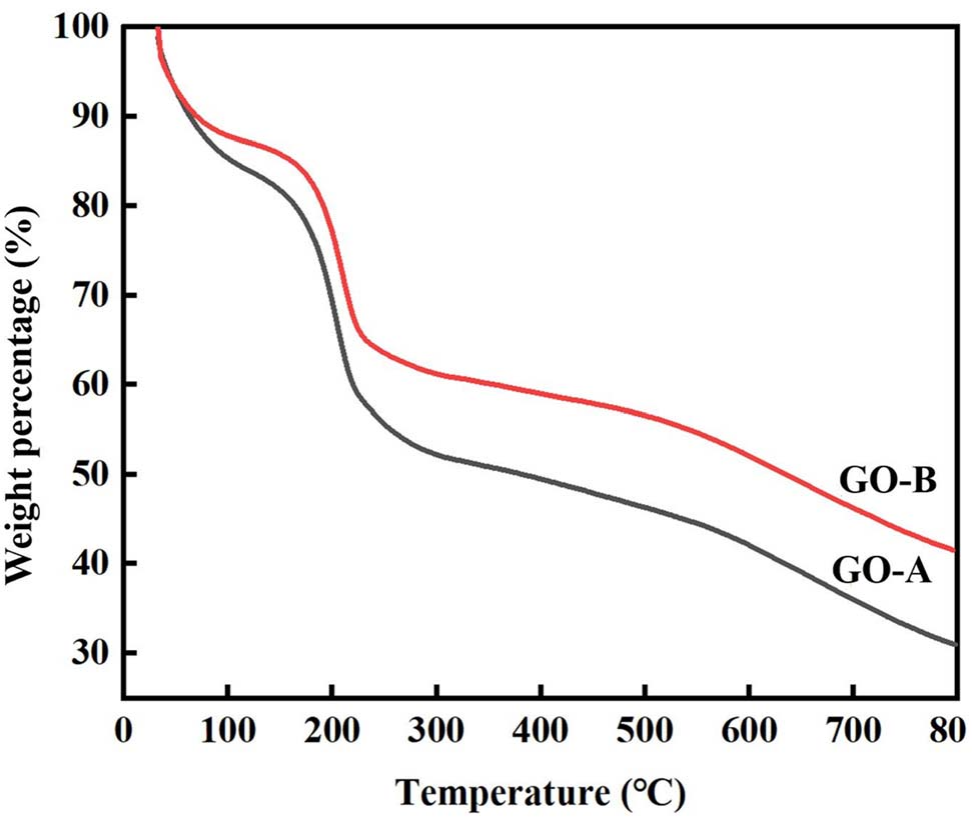
TGA curves of the GO-A and GO-B.

An independent expert has viewed the corrected image and has concluded that it is consistent with the discussions and conclusions presented.

The Royal Society of Chemistry apologises for these errors and any consequent inconvenience to authors and readers.

## Supplementary Material

